# Barrier Disrupting Effects of *Alternaria Alternata* Extract on Bronchial Epithelium from Asthmatic Donors

**DOI:** 10.1371/journal.pone.0071278

**Published:** 2013-08-23

**Authors:** Marina S. Leino, Matthew Loxham, Cornelia Blume, Emily J. Swindle, Nivenka P. Jayasekera, Patrick W. Dennison, Betty W. H. Shamji, Matthew J. Edwards, Stephen T. Holgate, Peter H. Howarth, Donna E. Davies

**Affiliations:** 1 Academic Unit of Clinical and Experimental Sciences and the Southampton NIHR Respiratory Biomedical Research Unit, University of Southampton Faculty of Medicine, Sir Henry Wellcome Laboratories, South Block, University Hospital Southampton, Southampton, United Kingdom; 2 Novartis Institutes for Biomedical Research, Novartis Horsham Research Centre, Horsham, United Kingdom; French National Centre for Scientific Research, France

## Abstract

Sensitization and exposure to the allergenic fungus *Alternaria alternata* has been associated with increased risk of asthma and asthma exacerbations. The first cells to encounter inhaled allergens are epithelial cells at the airway mucosal surface. Epithelial barrier function has previously been reported to be defective in asthma. This study investigated the contribution of proteases from *Alternaria alternata* on epithelial barrier function and inflammatory responses and compared responses of *in vitro* cultures of differentiated bronchial epithelial cells derived from severely asthmatic donors with those from non-asthmatic controls. Polarised 16HBE cells or air-liquid interface (ALI) bronchial epithelial cultures from non-asthmatic or severe asthmatic donors were challenged apically with extracts of *Alternaria* and changes in inflammatory cytokine release and transepithelial electrical resistance (TER) were measured. Protease activity in *Alternaria* extracts was characterised and the effect of selectively inhibiting protease activity on epithelial responses was examined using protease inhibitors and heat-treatment. In 16HBE cells, *Alternaria* extracts stimulated release of IL-8 and TNFα, with concomitant reduction in TER; these effects were prevented by heat-treatment of the extracts. Examination of the effects of protease inhibitors suggested that serine proteases were the predominant class of proteases mediating these effects. ALI cultures from asthmatic donors exhibited a reduced IL-8 response to *Alternaria* relative to those from healthy controls, while neither responded with increased thymic stromal lymphopoietin (TSLP) release. Only cultures from asthmatic donors were susceptible to the barrier-weakening effects of *Alternaria*. Therefore, the bronchial epithelium of severely asthmatic individuals may be more susceptible to the deleterious effects of *Alternaria*.

## Introduction

Asthma is a chronic inflammatory airways disease that is characterised physiologically by airway hyperresponsiveness to innocuous stimuli, and pathologically by Th2 inflammation and structural remodelling of the airways. The majority of asthma is associated with atopy, where an IgE response to specific aeroallergens has developed. The airway epithelium which forms both a physical barrier and an interface with the immune system *via* expression of adhesion molecules and secretion of a myriad cytokines, chemokines, and inflammatory mediators [1]–[3], is the first tissue encountered by such inhaled allergens. However, recent evidence has suggested that the asthmatic bronchial epithelium is structurally perturbed, resulting in impairment of barrier function [4].

Allergens from fungi including species of *Alternaria*, *Cladosporium*, and *Aspergillus* present a major risk factor for the development of asthma, with evidence supporting a link between sensitisation to fungi and prevalence or severity of asthma [5], [6], and also between spore prevalence and asthma [7], [8]. Further reports have found links between sensitivity to the common *Alternaria* species of fungi, particularly *Alternaria alternata*, and asthma [7], [9]–[12]. Pathogenic fungi secrete a range of proteases [13], with those of the serine class being particularly associated with asthma [14]. *In vitro* studies have shown that serine proteases from *Aspergillus fumigatus* and *Alternaria alternata* induce production of IL-8 and IL-6, as well as causing epithelial cell detachment [15]–[17]. This action is reported to occur *via* activation of protease activated receptor (PAR)−2 [18], [19], which has been observed to be up-regulated in the bronchial epithelium of asthmatic subjects [20], and can be replicated by use of a synthetic PAR-2 activating peptide [21]. Similar effects have been seen with the house dust mite (HDM) cysteine and serine protease allergens Der p 1 and Der p 3 [18], [22], and also serine proteases from German cockroach extract [23]. *Alternaria*-induced PAR-2 cleavage has also been suggested to be responsible for TSLP release from epithelial cells, and the activity of proteases as adjuvants in allergic sensitisation [24]–[26]. However, even though it is well recognised that the bronchial epithelium is a polarised structure, little is known about the directionality of epithelial cytokine release. Vectorial cytokine release may be critical for establishing concentration gradients of certain cytokines that play key roles in chemoattraction and immune cell recruitment.

Aside from their activity towards PARs, proteases can also perturb the epithelial barrier by directly cleaving tight junction proteins, facilitating permeation of allergens and pathogens to the underlying tissue. Der p 1 can degrade occludin and trigger ZO-1 breakdown, resulting in tight junction disassembly and increased paracellular permeability [27], [28]; similar effects have also been seen with HDM serine proteases [29]. The *Penicillium* allergen Pen ch 13, a serine protease, has been reported to cleave occludin [30], and effects on occludin, ZO-1, and claudin-1 have been noted with serine and cysteine proteases in pollen from a variety of sources [31].

Previous studies have focused on use of epithelial cell lines which are not covered by a cytoprotective mucous layer that protects the airways *in vivo*, which may explain their susceptibility to the proteolytic effects of allergen-derived proteases. Furthermore, the observation that there is impaired epithelial barrier function in asthma [4] led us to investigate the effect of *Alternaria* on epithelial barrier function, and to determine whether there is a differential response to *Alternaria* extract between fully differentiated cultures of primary human bronchial epithelial cells (PBECs) from healthy donors and those from asthmatic donors. As *Alternaria* has been observed to be both pro-inflammatory [16] and to induce development of a Th2-type response [32], [33], vectorial secretion of IL-8, TNFα, thymic stromal lymphopoietin (TSLP), IL-18 and IL-33 was analysed.

## Methods

### Ethics Statement

Ethical approval had been obtained from the Southampton local research ethics committee under the description “Pathophysiology of Airway Diseases such as Asthma and COPD”, Rec. No 05/Q1702/165, code MRC0268. All volunteers had provided their written informed consent, and all samples were anonymous-linked, with access to patient-identifiable data being available only to those with prior ethical approval.

### Cell Culture

The human bronchial epithelial cell-line, 16-HBE14o- (a gift from Professor D.C. Gruenert, San Francisco, USA) [34] was cultured in Minimal Essential Medium (MEM) with GlutaMax supplemented with 10% heat-inactivated FBS, 50 IU/ml penicillin and 50 μg/ml streptomycin (all Invitrogen, Paisley, UK). Cells were placed in Transwell® culture inserts (Corning, Tewkesbury, MA, USA) pre-coated with collagen I (Pure-Col, Nutacon BV, Leimuiden, The Netherlands); they were used for experiment when the transepithelial electrical resistance (TER) was >3000 Ω/cm^2^. 24 h prior to challenge, 16HBE cells had the apical medium replaced with serum-free MEM.

Primary bronchial epithelial cells (PBECs) were grown from bronchial brushings obtained from non-asthmatic or severe asthmatic volunteers by fibreoptic bronchoscopy, as previously described [35] (for donor information, see Table S1). PBECs were expanded in culture and, at passage 2, were taken to an air-liquid interface (ALI) [4]. Cultures were used for assays at day 21 when the TER was >3000 Ω/cm^2^. 24 h prior to challenge, the basolateral medium of ALI cultures was replaced with BEBM containing 1% ITS (Sigma-Aldrich, Gillingham, UK) and 1.5 μg/ml BSA basolaterally.

Lyophilised *Alternaria alternata* and *Cladosporium herbarum* extracts (Greer, Lenoir, NC, USA) were prepared by purification and lyophilisation of fungal culture medium according to current Good Manufacturing Practice to minimise inter-batch variability [36]. Extracts were dissolved into supplement-free medium and added apically to cultures to achieve the desired concentrations. Protein concentrations were 136 µg/mg dry weight for *Alternaria* extract and 30 µg/mg dry weight for *Cladosporium* extract. LPS content was not assayed, however, previous assays of the same product have shown minimal endotoxin activity [32]; in view of the lack of CD14 expression by epithelial cells [37], there is minimal possibility that LPS contamination contributed to responses of the BEC cultures. To assess heat-lability, fungal extracts were heat-treated at 65°C for 30 min while the contribution of serine, cysteine and aspartate protease activity was determined by pretreating *Alternaria* extracts with AEBSF, E-64, or Pepstatin A (Sigma-Aldrich, Gillingham, UK) respectively for 30 min immediately prior to stimulation. To assess the contribution of p38 MAPK, SB203580 (Sigma-Aldrich) was added apically for 30 min prior to challenge. At 24 h, apical and basolateral media were harvested and cells were fixed for immunostaining. Further details of cell culture and challenge can be found in Information S1.

### Lactate Dehydrogenase (LDH) Assay

LDH release was measured using a CytoTox 96 LDH assay kit (Promega, Southampton, UK) according to the manufacturer's instructions. Total cellular LDH activity was determined by lysing cells with 1% Triton X-100 in culture medium for 60 min at 37°C.

### FITC-Dextran Passage

FITC-dextran (4 kDa) was added to the apical compartment at a final concentration of 2 mg/ml, 1h after the addition of the fungal extracts. Basolateral FITC-dextran concentration at 24 h was determined against a standard curve using a Labsystems Fluoroskan FL fluorimeter (Thermo Fisher Scientific, Waltham, MA), with excitation and emission wavelengths set to 485 nm and 530 nm respectively.

### Cytokine Analysis

Release of IL-8, TNFα, IL-18 and IL-33 into the apical and basolateral compartments was assayed by ELISA according to the manufacturer's instructions (R&D Systems, Abingdon, UK). For assay of TNFα release from ALI cultures, a high-sensitivity TNFα ELISA kit (R&D Systems) was used. TSLP was measured using an ‘in-house’ ELISA developed by Novartis Plc (Horsham, UK), which recognises both *E. coli* expressed recombinant TSLP and naturally secreted TSLP from primary lung fibroblasts.

### Detection of Protease Activity

*Alternaria* extract protease activity was assessed using a protease fluorescent detection kit (Sigma-Aldrich) according to the manufacturer's instructions. This kit was also used to measure attenuation of *Alternaria* protease activity by prior heat-treatment of the extract or the protease inhibitors AEBSF, E-64, or Pepstatin A.

### Statistical Analysis

Results were analysed by one-way repeated measures ANOVA with Bonferroni's correction for pairwise analyses, or a ranked version thereof (Friedman Repeated Measures ANOVA), with Bonferroni's or Tukey's correction for pairwise analyses, as appropriate. All analyses were performed using SigmaPlot 11.0 (Systat Software, Hounslow, UK).

## Results

### The Effect of *Alternaria* Extracts on Inflammatory Mediator Release by 16HBE Cells

**Figure 1:**
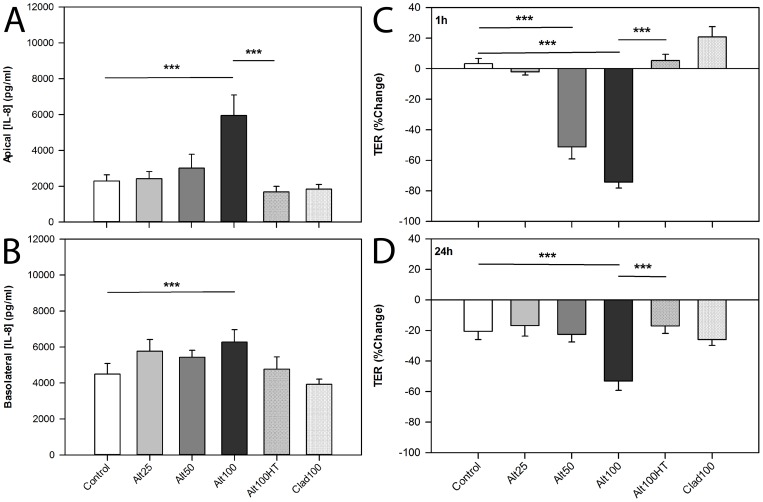
*Alternaria* extract induces a heat-labile increase in IL-8 release and rapid reduction in TER in polarised 16HBE cells. Polarised 16HBE cells on Transwell inserts were challenged apically with varying doses of *Alternaria* (Alt) or *Cladosporium* (Clad) fungal extracts, or heat treated fungal extract. (A) Apical and (B) basolateral supernatants were harvested 24 h post-challenge. IL-8 concentration was determined by ELISA (n = 4–9). TER was measured before challenge and at (C) 1h and (D) 24 h thereafter, and calculated as percentage change from pre-challenge readings (n = 4–15). Bars represent mean ± SEM. Analysis by one way repeated measures ANOVA with Bonferroni correction for pairwise analyses *** p<0.001.

Polarised 16HBE cells were challenged with *Alternaria* extract, and cytokine release after 24 h stimulation was measured by ELISA. *Alternaria* induced a dose-dependent increase in apical IL-8 release (Figure 1A), with 100 μg/ml *Alternaria* (Alt100) significantly inducing IL-8 release compared with untreated controls, or cells treated with heat-treated Alt100 (Alt100HT) or 100 μg/ml *Cladosporium* extract (Clad100). While Alt100 also induced a significant increase in basolateral IL-8 release compared to control (Figure 1B), this was less marked than apical release. Clad100 did not increase basolateral IL-8 release above baseline and heat treatment of the *Alternaria* extract (Alt100HT) tended to reduce the stimulation of basolateral IL-8 release, but this failed to reach statistical significance. Analysis of TNFα release after *Alternaria* challenge revealed similar results to IL-8 analysis (Figure S1).

### The Effect of *Alternaria* on 16HBE Transepithelial Electrical Resistance

We next investigated the effect of *Alternaria* on TER. A significant dose-dependent decrease in TER was observed 1h post-challenge with both Alt50 and Alt100 (Figure 1C). With Alt50, the drop in TER began to recover almost immediately, particularly from 3 h onwards, reaching control levels by 6 h (Figure S2). After challenge with Alt100, the TER remained significantly lower than control, even 24 h post-challenge (Figure 1D). As found with cytokine release, Alt100HT showed no significant effect on TER at any time point. Similarly, Clad100 did not affect TER. The dose-dependent, heat-labile increase in epithelial permeability caused by *Alternaria* extracts was confirmed by studies with FITC-labelled 4 kDa dextran (Figure S3), suggesting that *Alternaria* affects both ionic and macromolecular permeability; however the extent to which passage of the 4 kDa macromolecule was facilitated by exposure of the epithelium to *Alternaria* was small compared to the effect of the calcium chelator EGTA (50 μM). None of the challenges significantly induced LDH release compared with control cultures (data not shown).

### Protease Activity of *Alternaria* Extract

**Figure 2:**
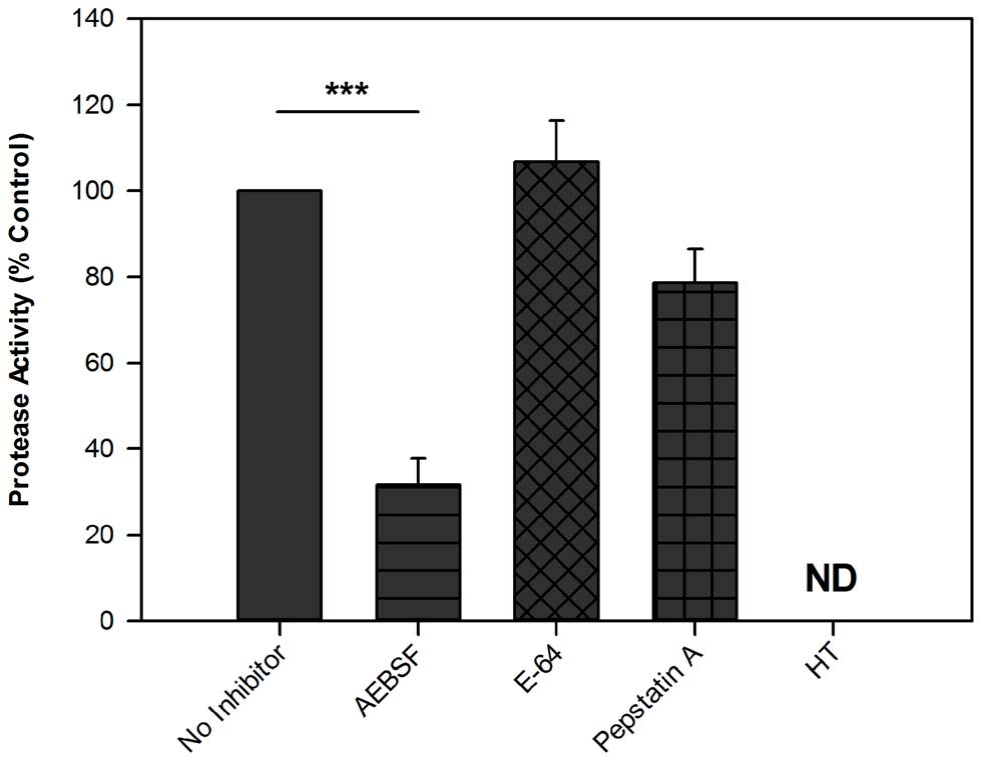
Protease activity of *Alternaria* extract is reduced by the serine protease inhibitor AEBSF. FITC-labelled casein was incubated with *Alternaria* extract (500 μg/ml) alone or in the presence of either AEBSF (2.5 mM), E-64 (500 μM), Pepstatin A (5 μg/ml), or heat-treated (n = 4 separate experiments measured in duplicate). Soluble fluorescence was measured after 24 h, relative to a trypsin standard. Bars represent mean fluorescence relative to inhibitor-free control±SEM. Protease activity of 100 µg/ml Alternaria extract was equivalent to 6.7±2.5 µg/ml trypsin; for comparison, Cladosporium protease activity was equivalent to 3.3±0.0 μg/ml trypsin. Analysis as for Figure 1. Bars represent mean ± SEM; *** p<0.001.

To determine specific protease activity in *Alternaria* extract, a fluorescent protease assay was performed in the presence of a variety of protease inhibitors (Figure 2). The serine protease inhibitor AEBSF significantly reduced the protease activity of *Alternaria* extract whereas the aspartate protease inhibitor Pepstatin A had a small effect, and the cysteine protease inhibitor E-64 had no effect. Heat-treatment of *Alternaria* extract reduced protease activity to below the detection limit of the assay. No inhibitor possessed intrinsic proteolytic activity.

### The Effect of Protease Inhibitors on Proinflammatory Cytokine Release

**Figure 3:**
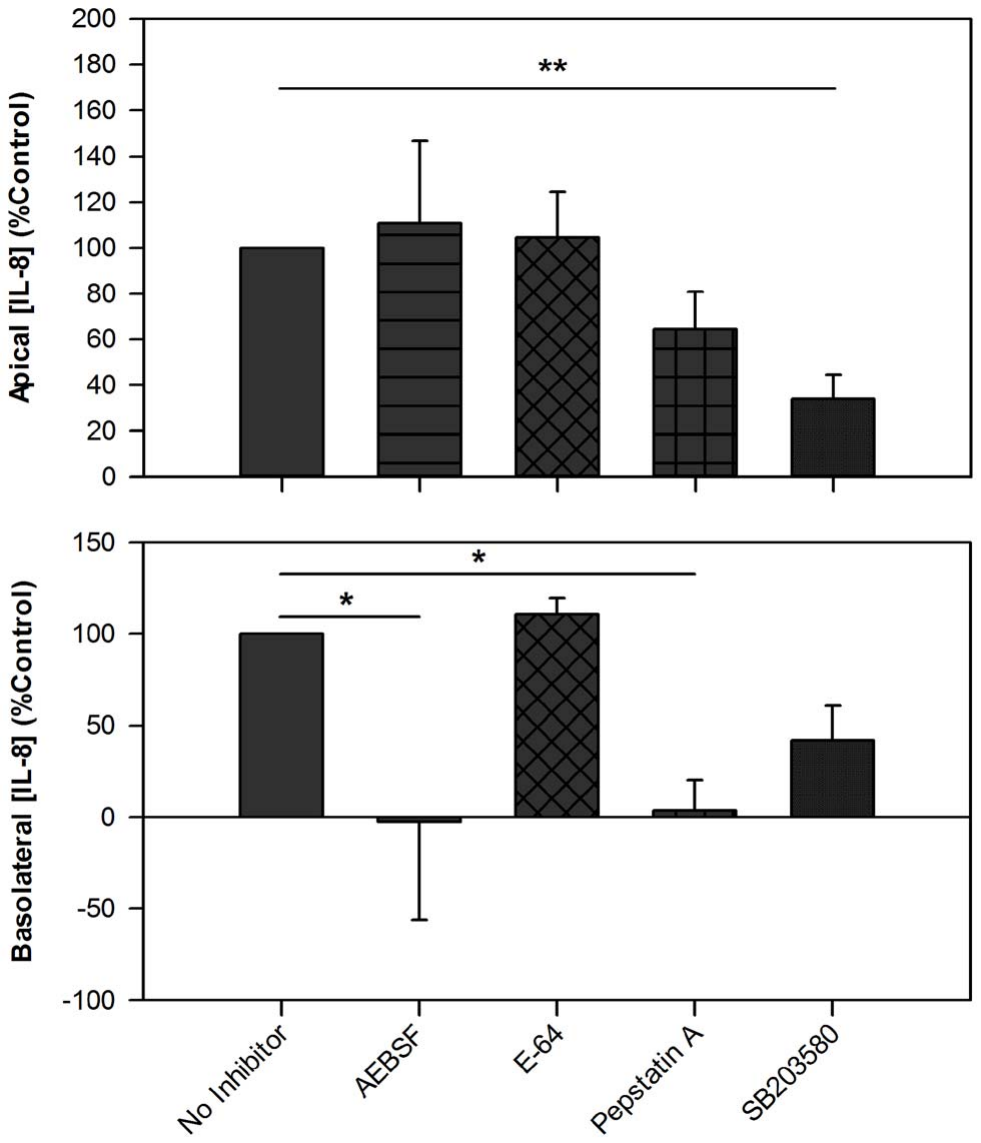
Inhibitors of proteases and p38 MAPK differentially inhibit apical and basolateral IL-8 release after *Alternaria* challenge. The effect of *Alternaria* extract (100 μg/ml) on 16HBE cells was tested alone or in the presence of AEBSF (250 μM), E-64 (50 μM), Pepstatin A (0.5 μg/ml) or SB203580 (25 μM) (n = 3–8). IL-8 release 24 h post-challenge was calculated as “Release (% control)  =  ((Alt_INHIB_ – No Alt_INHIB_)/(Alt_NO_ _INHIB_ – No Alt_NO INHIB_)) ×100”, to correct for any effect of the inhibitors on baseline IL-8 release without *Alternaria*. Data show mean ± SEM. Analysis as for Figure 1; * p<0.05, ** p<0.01.

To examine whether protease activity contributed to the effect of *Alternaria* on the epithelial cells, Alt100 was pre-incubated with protease inhibitors prior to challenge of the epithelial cultures; control cultures were tested in the presence of inhibitor alone. Apical release of IL-8 in response to Alt100 was not significantly affected by any of the protease inhibitors; in contrast both AEBSF and Pepstatin A significantly reduced basolateral IL-8 release (Figure 3). As we failed to affect apical cytokine release with protease inhibitors, we also tested the effect of inhibiting p38 MAPK which we have previously found to inhibit pollen-induced apical IL-8 release, without affecting transcription [38]. Correspondingly, the present data shows SB203580 significantly reduced apical IL-8 release while not significantly affecting basolateral cytokine release. Neither apical nor basolateral release of TNFα were significantly affected by any of the inhibitors tested (Figure S4).

### The Effect of Protease Inhibitors on the Permeabilising Activity of *Alternaria*

To further explore the effect of protease inhibition, TER changes were investigated using Alt100 pre-treated with the same inhibitors. At 1h post-challenge, either E-64 or SB203580 significantly inhibited the decrease in TER caused by *Alternaria* (Figure S5). At 24 h post-challenge, while inter-treatment differences approached significance, the significance threshold was not crossed. However, there was a notable attenuation of the effect of *Alternaria* on TER after incubation with either AEBSF or Pepstatin A.

### The Effect of *Alternaria* on Polarised Cytokine Release from ALI Cultures

Having observed significant effects of *Alternaria* using the 16HBE bronchial epithelial cell line, experiments were performed using primary epithelial cells differentiated at an ALI. For these experiments, cells were derived from non-asthmatic and severe asthmatic donors allowing disease-related comparisons to be made. Preliminary studies showed that higher concentrations of *Alternaria* were required to elicit responses in ALI cultures compared to 16HBE, and therefore up to 400 μg/ml was used.

**Figure 4:**
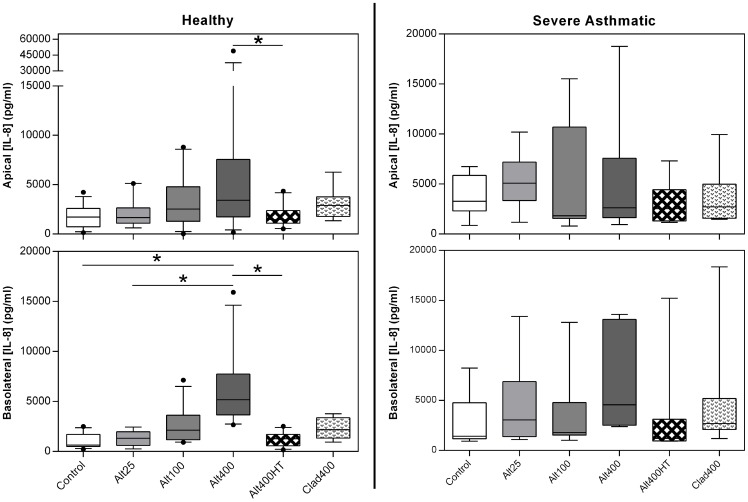
ALI cultures of healthy, but not severely asthmatic, donors increase IL-8 release in response to *Alternaria*. ALI cultures from healthy (n = 8–12) or severely asthmatic (n = 6–7) donors were differentiated at air-liquid interface, prior to challenge with *Alternaria* (Alt) or *Cladosporium* (Clad) fungal extracts. IL-8 release 24 h post-challenge was determined by ELISA. Box shows median and 25^th^/75^th^ percentiles, and whiskers show 10^th^/90^th^ percentiles. Analysis by Friedman's test with Tukey's correction for pairwise analyses; * p<0.05.

In ALI cultures from healthy donors, the highest dose of *Alternaria* (Alt400) significantly induced an approximate doubling of IL-8 release apically *versus* heat-treated *Alternaria* (Figure 4). *Alternaria* also tended to cause a dose-dependent increase in basolateral release of IL-8, significant for Alt400 with IL-8 levels eight-fold higher than control. *Cladosporium* extract had no significant effect on IL-8 release. Thus, basolateral IL-8 release tended to be more responsive to *Alternaria* than was apical IL-8 release (see Figure S6), the reverse of the situation seen in 16HBE cells, although the degree of inter-donor variability makes it difficult to draw robust conclusions regarding this directionality. When the same experiments were performed on ALI cultures from severely asthmatic donors, none of the challenges elicited a significant increase in release of IL-8 into either compartment (Figure 4). TNFα levels were generally below the lower detection limit (0.5 pg/ml) of the high-sensitivity ELISA kit.

Given the previous association of *Alternaria* with development of a Th2 phenotype [32], [33], [39], we also assessed release of IL-18, IL-33, and TSLP by ALI cultures after exposure to *Alternaria*. Using commercially available ELISA kits, these cytokines were undetectable (lower limit of detection  = 10.2 pg/ml) in either apical or basolateral supernatants across all groups. However, using an ‘in-house’ TSLP ELISA, basolateral TSLP release was detectable but there was no significant difference in TSLP secretion comparing untreated or *Alternaria*-stimulated ALI cultures from either healthy or severe asthmatic donors (Figure S7). No apical secretion of TSLP was detectable.

### The Effect of *Alternaria* on ALI Culture Transepithelial Electrical Resistance

**Figure 5:**
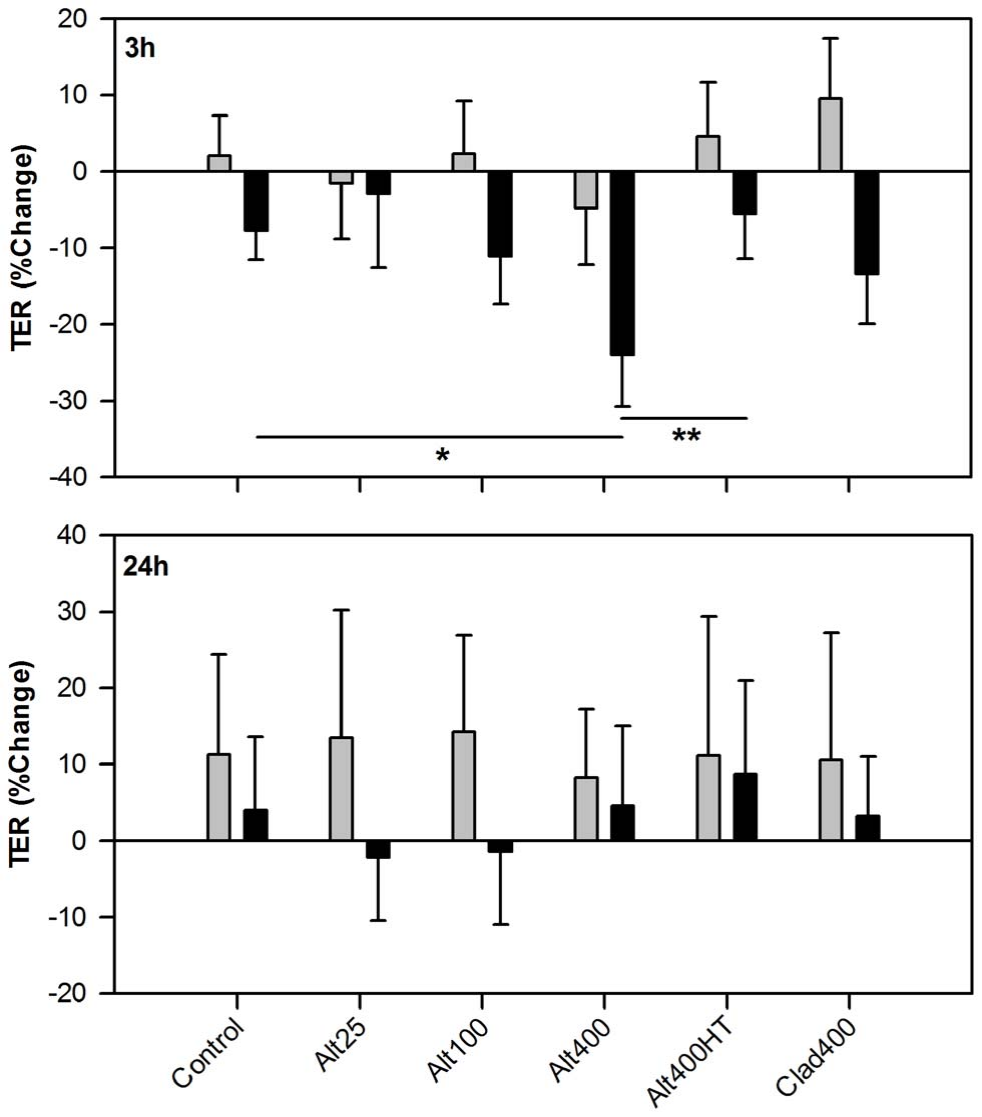
ALI cultures from severely asthmatic donors are sensitive to the barrier weakening effect of *Alternaria*. ALI cultures from healthy (grey bars; n = 7–9) and severely asthmatic (black bars; n = 6–7) donors were challenged with *Alternaria* (Alt) or *Cladosporium* (Clad) fungal extracts. TER was measured pre-challenge and 3 h and 24 h post-challenge. Results are expressed as percentage change in TER relative to pre-challenge and are shown as mean ± SEM. Analysis as for Figure 1; * p<0.05, ** p<0.01.

In ALI cultures from healthy donors, *Alternaria* had no effect on TER after 3 h (Figure 5). By 24 h, TERs in the healthy donor ALIs were all increased to 8–14% of their baseline levels, with no difference between challenges. In contrast, cultures derived from severely asthmatic donors responded to *Alternaria* challenge with a rapid dose-dependent decrease in TER at 3 h compared to control and heat-treated *Alternaria*. By 24 h post-challenge, no difference existed between treatments. These results suggest that, unlike ALI cultures from healthy donors, asthmatic donor ALI cultures are susceptible to a rapid loss of epithelial barrier function after exposure to *Alternaria*. The heat lability of this effect suggests that it is protease-mediated.

## Discussion

This study investigated the potential of *Alternaria* proteases to perturb airway epithelial barrier function using cultures that are fully differentiated and covered by a layer of cytoprotective mucus as occurs *in vivo*, and to determine whether these responses are different in severe asthma. Our main findings were that ALI cultures from severely asthmatic donors exhibited a more variable IL-8 response to *Alternaria* extracts relative to those from healthy donors, while only cultures from severely asthmatic donors were susceptible to the barrier-weakening effects of *Alternaria*. Furthermore, while previous studies with cell lines have suggested that exposure to *Alternaria* extracts leads to production of cytokines that promote a Th2 response [33], [39], we failed to detect any epithelial release of IL-33 or IL-18, while basolateral TSLP secretion did not change in response to *Alternaria*.

Induction of inflammation has previously been shown to occur upon challenge with serine proteases from *Aspergillus fumigatus* [15] and *Alternaria* [40], [41]. However, these studies did not take into account epithelial polarisation, and were thus unable to study the directionality of cytokine release, which is important in the context of establishing a chemotactic gradient for IL-8 to act as a neutrophil chemoattractant [42]–[45]. The results of this study also emphasise the importance of cell differentiation since *Alternaria*-induced release of IL-8 and TNFα was predominantly apical for undifferentiated 16HBE cells, whereas for differentiated ALI cultures the greatest fold stimulation was observed for the basolateral compartment (median 2.9 fold apical *versus* 7.1 fold basolateral in healthy donor cultures). Also of note is the observation that TSLP is exclusively released into the basolateral compartment highlighting the importance of epithelial polarisation for vectorial cytokine secretion. This may be significant in terms of establishing appropriate concentration gradients for chemoattractants that direct cells to the luminal or subepithelial compartments.

Although *Alternaria*-stimulated IL-8 release into the basolateral compartment was relatively small, this stimulatory effect was greatly inhibited by AEBSF and Pepstatin A suggesting a protease-mediated effect. Conversely, protease inhibition failed to affect apical IL-8 release, which was instead sensitive to inhibition of p38 MAPK which is thought to play a role in stabilising IL-8 mRNA [46]. Although it has been well established that some growth factors are selectively sorted to the basolateral surface *via* information that usually resides in the cytoplasmic tail of the cargo [47], this does not explain how trafficking of a protein such as IL-8 is differentially regulated towards the apical or basolateral domains. Although this question has not been explored in detail, we have recently shown that trafficking of IL-8 in ALI cultures challenged with grass pollen extract is post-transcriptionally regulated with apical release being selectively regulated by p38 MAPK [38] as observed in the present study using *Alternaria*. While the PAR-2 agonist trypsin, as well as a PAR-2 activating peptide, have been shown to activate p38 MAPK expression weakly, it seems unlikely that PAR-2 activation lies upstream of p38 MAPK activation in mediating *Alternaria*-induced apical IL-8 release as this response was insensitive to protease inhibitors.

Although HDM serine and cysteine proteases have been reported to increase epithelial permeability [27], [29], there is a paucity of comparative studies of fungal proteases, particularly using *Alternaria*. The present study suggests that heat labile activities in *Alternaria* extracts significantly and rapidly weaken the epithelial barrier, initially due to cysteine protease activity, and also *via* a p38 MAPK-mediated mechanism. However, both E-64 and SB203580 exhibited non-specific barrier-weakening effects at the 1h time point, and this may be a factor in the apparent reduction of activity of *Alternaria* in this respect. At 24 h a lesser degree of non-specific barrier-weakening activity was displayed by the inhibitors, and serine and aspartate protease inhibition appeared to show a trend for inhibition of the reduction in TER caused by *Alternaria*.

Fungal proteases fulfil a number of functions, particularly the external digestion of macronutrients, but are also intrinsic to the pathogenesis of diseases resulting from fungal exposure [48]. Taken together, these results suggest that the effects of *Alternaria* at the airway epithelium are due to serine and, to a lesser extent, aspartate protease activity, with the involvement of proteases in general being supported by the lack of activity of heat-treated *Alternaria* extract. Recent reports have suggested a role for serine and aspartate proteases in the cellular effects of *Alternaria* but, to our knowledge, this is the first work which suggests that both classes of protease exert significant effects. For example, it has been suggested that the active component(s) of *Alternaria* in the induction of proinflammatory responses in bronchial epithelial cells is serine protease(s) acting on PAR-2 [40], although this has not been confirmed *in vivo* [49]. In contrast, it has been suggested recently that the actions of *Alternaria* on epithelial cells and eosinophils are sensitive to aspartate protease inhibition, but not to serine protease or cysteine protease inhibition [50]–[52]. In our studies, serine proteases were the dominant activity in the protease assays, although inhibition of either serine or aspartate protease activity had a potent suppressive effect on IL-8 release. This discrepancy may be explained by the relative sensitivity of the fluorescent assay for serine and aspartate proteases or alternatively, it is possible that the effect of Pepstatin A on IL-8 release may be due, in part, to inhibition of endogenous proteases. For example, it has been reported that challenge of epithelial cells with *Aspergillus fumigatus* triggers release of lysosomal enzymes including the aspartate protease, cathepsin D [53], which may affect subsequent cytokine responses. In addition to proteases, other constituents of *Alternaria* extract such as β-glucans and chitin (cell wall components) have been noted to exert immunomodulatory effects [54]–[56]. However, the lack of residual effect after heat-treatment of *Alternaria* in the present study suggests that the effect of β-glucans was negligible in our studies, although we cannot exclude the effect of other unidentified components, either proteinaceous or small molecules, which may be affected by heat treatment.

The study was then extended to examine primary bronchial epithelial cells in ALI cultures, as a more accurate model of human airway exposure. ALI cultures from non-asthmatic donors exhibited increased IL-8 release in response to *Alternaria*, while responses of cultures from asthmatic donors exhibited a trend of being blunted and not statistically significant, although considerable inter-individual variability was observed.

ALI cultures from healthy donors were resistant to the increase in epithelial permeability seen when ALI cultures from severely asthmatic donors were challenged with *Alternaria*. To our knowledge, this is the first study which has examined differential responses to *Alternaria* between healthy and asthmatic ALI cultures. The implications are potentially significant: if the permeability of the bronchial epithelium in asthma is significantly increased by *Alternaria*, passage of inhaled allergens to the subepithelial tissue may be facilitated, and epithelial homeostasis disrupted. Since ALI cultures from asthmatic donors also displayed a lack of response to *Alternaria* in terms of IL-8 secretion, the ability to upregulate recruitment of neutrophils and clearance of allergens and toxicants in response to *Alternaria* may be affected. This impaired response of the epithelial cells from asthmatic donors to *Alternaria* is unlikely to be due to carry-over of corticosteroids used for asthma control therapy, as we have shown that similar ALI cultures respond to pollen extract with a significant increase in IL-8 release irrespective of whether they were derived from healthy or severely asthmatic donors [38].

Using commercially available ELISA kits, we failed to detect any IL-18, IL-33 or TSLP release by the ALI cultures. IL-33 release has been shown to be increased by *Alternaria* in murine BAL fluid and in normal human bronchial epithelial cells (NHBE) [33], however in the latter case the epithelial cell cultures were undifferentiated. In contrast, others have failed to detect IL-33 or TSLP secretion from either NHBEs challenged with *Alternaria* [39] or mouse lung epithelial cells challenged with *Aspergillus fumigatus* [56]. We also failed to detect IL-18, despite a recent report of a marked rapid release of IL-18 after *Alternaria* challenge of NHBE cells [39]. However, control experiments performed in our laboratory and assayed at the same time showed IL-18 and TSLP production in response to rhinovirus challenge (data not shown), suggesting that the lack of any observable effect with *Alternaria* was not due to defective epithelial synthesis or release of these mediators. Although we initially failed to detect TSLP with a commercial ELISA (from R&D Systems), use of an alternative ‘in-house’ ELISA (developed by Novartis plc.) enabled detection and quantification of basolateral TSLP release, however no apical secretion was evident. We postulate that this difference in detection is due to differences in antibody recognition of recombinant and native TSLP, making the commercial ELISA kit much less sensitive for detection of the naturally produced protein. Having observed such a large difference in sensitivity for detection of TSLP, it is possible that the ELISAs employed for measurement of IL-33 and IL-18 may also be similarly compromised.

TSLP is secreted by epithelial cells and potently activates human dendritic cells to release a battery of cytokines which results in Th2-skewing of naïve CD4+ T cells [57]. *Alternaria* has been shown to increase TSLP expression and release from airway epithelial cells *in vitro via* PAR-2 activation [26]. However, even though we were confident in our ability to detect TSLP reliably, we found no significant change in TSLP release in response to *Alternaria*. One likely explanation is the use of fully differentiated ALI cultures in the present work, as opposed to undifferentiated monolayers in the previous studies, emphasising the importance of using models that closely mimic the *in vivo* state. While there is strong evidence to suggest that *Alternaria* exposure can induce Th2-type responses *in vivo*, and that TSLP released from structural cells can act as a potent mediator for Th2 skewing [58], it is possible that epithelial cells require the presence of other cell types or multiple stimuli for *Alternaria* to have such an effect. For example, IL-4 and double-stranded RNA potently synergise to stimulate TSLP release from bronchial epithelial cells *in vitro* [59].

In summary, this study demonstrates that *Alternaria* extract is able to significantly induce release of inflammatory cytokines and to increase the permeability of a polarised airway epithelial cell line. These effects were attributed to a heat-labile component of the *Alternaria*, identified as being serine and possibly aspartate protease mediated. Crucially, this study is the first to demonstrate that fully differentiated epithelial cultures from asthmatic donors appear to have a blunted IL-8 response to high levels of *Alternaria*, while at the same time being more susceptible to the barrier-weakening effect of *Alternaria*, than those from healthy donors.

## Supporting Information

Figure S1 **Alternaria extract induces a heat-labile increase in TNFα release from polarised 16HBE cells.** Polarised 16HBE cells on Transwell inserts (n = 3–9) were challenged apically with *Alternaria* (Alt) or *Cladosporium* (Clad) fungal extracts. Apical and basolateral supernatants were harvested 24 h post-challenge. TNFα concentration was determined by ELISA. Analysis by one way repeated measures ANOVA with Bonferroni correction for pairwise analyses. Bars represent mean ± SEM; ** p<0.01; *** p<0.001.
(TIF)

Figure S2 ***Alternaria*** **extract induces a dose-dependent decrease in 16HBE TER.** TER was measured before fungal challenge of polarised 16HBE cells, and at regular intervals up to 24 h thereafter (n = 4–15). Graph shows TER of polarised 16HBE cultures in medium alone (▴), or with *Alternaria* extract at 50 (•) and 100 μl/ml (♦), expressed as percentage change from pre-challenge value. Points represent mean ± SEM.
(TIF)

Figure S3 ***Alternaria*** **extract increases epithelial macromolecular permeability.** Polarised 16HBE cells on Transwell inserts were challenged with medium, Alt50, Alt100 (all n = 4), Alt100HT, Clad100, or EGTA (n = 2) 1 h before addition of 2 mg/ml 4 kDA FITC-dextran. After 24 h challenge, basolateral FITC-dextran concentration was determined fluorimetrically. Analysis as for Figure S1 Bars represent mean ± SEM; ** p<0.01.
(TIF)

Figure S4 **Inhibitors of proteases and p38 MAPK have no significant effect on apical or basolateral TNFα release after fungal challenge.** The effect of *Alternaria* (100 μg/ml) on 16HBE cells was tested alone or in the presence of AEBSF (250 μM), E-64 (50 μM), Pepstatin A (0.5 μg/ml) or SB203580 (25 μM) (n = 3–8). TNFα release 24 h post-challenge was calculated as “Release (% control)  =  ((Alt_INHIB_ – No Alt_INHIB_)/(Alt_NO_ _INHIB_ – No Alt_NO INHIB_)) ×100”, to correct for any effect of the inhibitors on baseline TNFα release without *Alternaria*. Analysis as for Figure S1. Data show mean ± SEM.
(TIF)

Figure S5 ***Alternaria*** **-induced drop in TER is sensitive to inhibition of cysteine protease and p38 MAPK.** The effect of *Alternaria* (100 μg/ml) on 16HBE cells was tested alone or in the presence of AEBSF (250 μM), E-64 (50 μM), Pepstatin A (0.5 μg/ml) or SB203580 (25 μM) (n = 3–6). TER was measured at 1 h and 24 h post-challenge, calculated as percentage change from pre-challenge, and corrected for any effect of the inhibitor alone by subtracting the percentage change in TER in the absence of Alternaria from the percentage change in TER in the presence of Alternaria, with each respective inhibitor or inhibitor-free condition. Bars represent mean change ± SEM; ** p<0.01.
(TIF)

Figure S6 **The increase in IL-8 release in healthy donor ALI cultures is driven by increased basolateral release of IL-8.** ALI cultures from healthy (n = 8–12) or severely asthmatic (n = 6–7) donors were differentiated at air-liquid interface, prior to challenge with Alternaria (Alt) 400 µg/ml. IL-8 release 24 h post-challenge was determined by ELISA. Lines represent difference in individual donor cultures between control and Alt400-stimulated IL-8 release. Analysis by Wilcoxon Matched Pair test. *** p<0.001.
(TIF)

Figure S7 **Alternaria challenge does not affect basolateral release of TSLP in healthy or severely asthmatic donor ALI cultures.** ALI cultures from healthy (n = 7–12) or severely asthmatic (n = 6–7) donors were differentiated at air-liquid interface, prior to challenge with *Alternaria* (Alt) or *Cladosporium* (Clad) fungal extracts. TSLP release 24 h post-challenge was determined by ELISA. TOP: Boxes show median and 25/75^th^ percentiles, and whiskers show 10^th^/90^th^ percentiles. Analysis by Friedman's test. BOTTOM: Lines represent difference in individual donor cultures between control and Alt400-stimulated TSLP release.
(TIF)

Information S1 (DOCX)

Table S1 **PBEC donor information.** Clinical characterisation of the donors of the bronchial epithelial cells used in this work. FEV_1_% – forced expiratory volume in 1 second, as a percentage of predicted value; ICS – inhaled corticosteroid (dose as equivalent to micrograms per day Beclometasone dipropionate); LABA  =  long acting β_2_-adrenoceptor agonist; anti-leuk  =  anti-leukotriene.
(DOCX)
