# Supplementary figures and images for: Barrier Disrupting Effects of Alternaria Alternata Extract on Bronchial Epithelium from Asthmatic Donors
Source: PLoS One. 2013 Aug 23;8(8):e71278. doi: 10.1371/journal.pone.0071278 (PMC3751915; doi:10.1371/journal.pone.0071278)

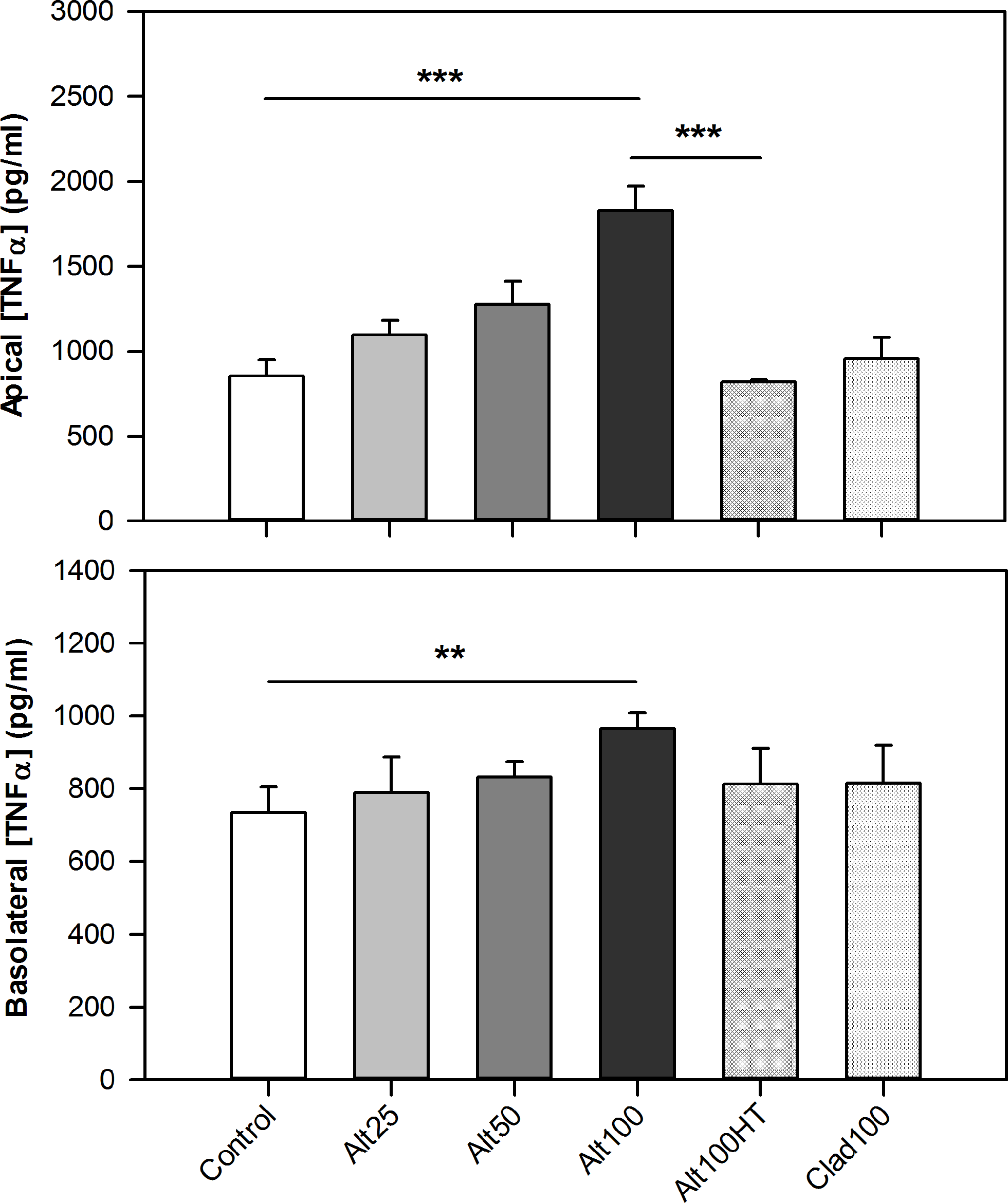

Supplement: Figure S1 — Alternaria extract induces a heat-labile increase in TNFα release from polarised 16HBE cells. Polarised 16HBE cells on Transwell inserts (n = 3–9) were challenged apically with Alternaria (Alt) or Cladosporium (Clad) fungal extracts. Apical and basolateral supernatants were harvested 24 h post-challenge. TNFα concentration was determined by ELISA. Analysis by one way repeated measures ANOVA with Bonferroni correction for pairwise analyses. Bars represent mean ± SEM; ** p<0.01; *** p<0.001. (TIF) [file pone.0071278.s001.tif]

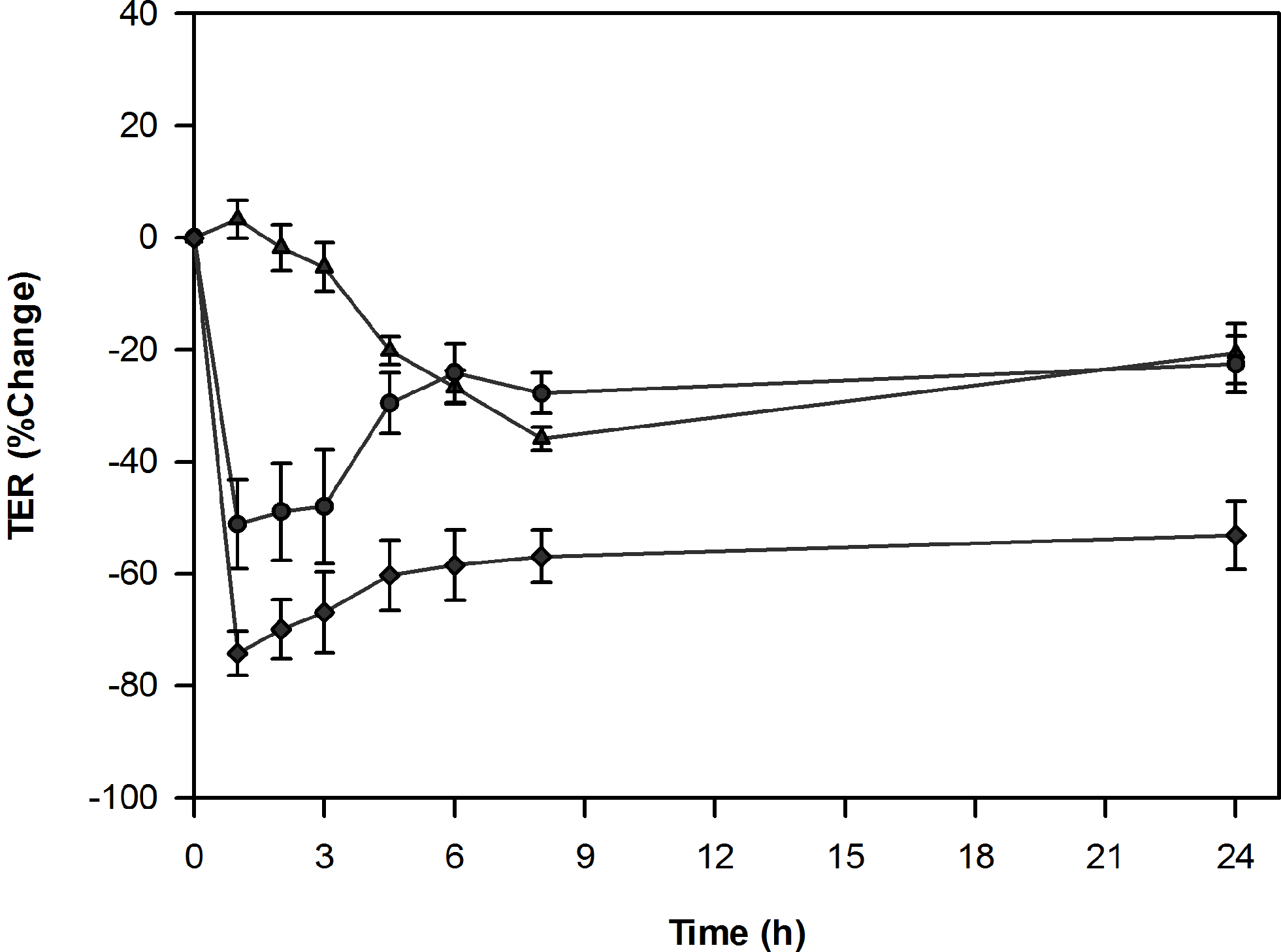

Supplement: Figure S2 — Alternaria extract induces a dose-dependent decrease in 16HBE TER. TER was measured before fungal challenge of polarised 16HBE cells, and at regular intervals up to 24 h thereafter (n = 4–15). Graph shows TER of polarised 16HBE cultures in medium alone (▴), or with Alternaria extract at 50 (•) and 100 μl/ml (♦), expressed as percentage change from pre-challenge value. Points represent mean ± SEM. (TIF) [file pone.0071278.s002.tif]

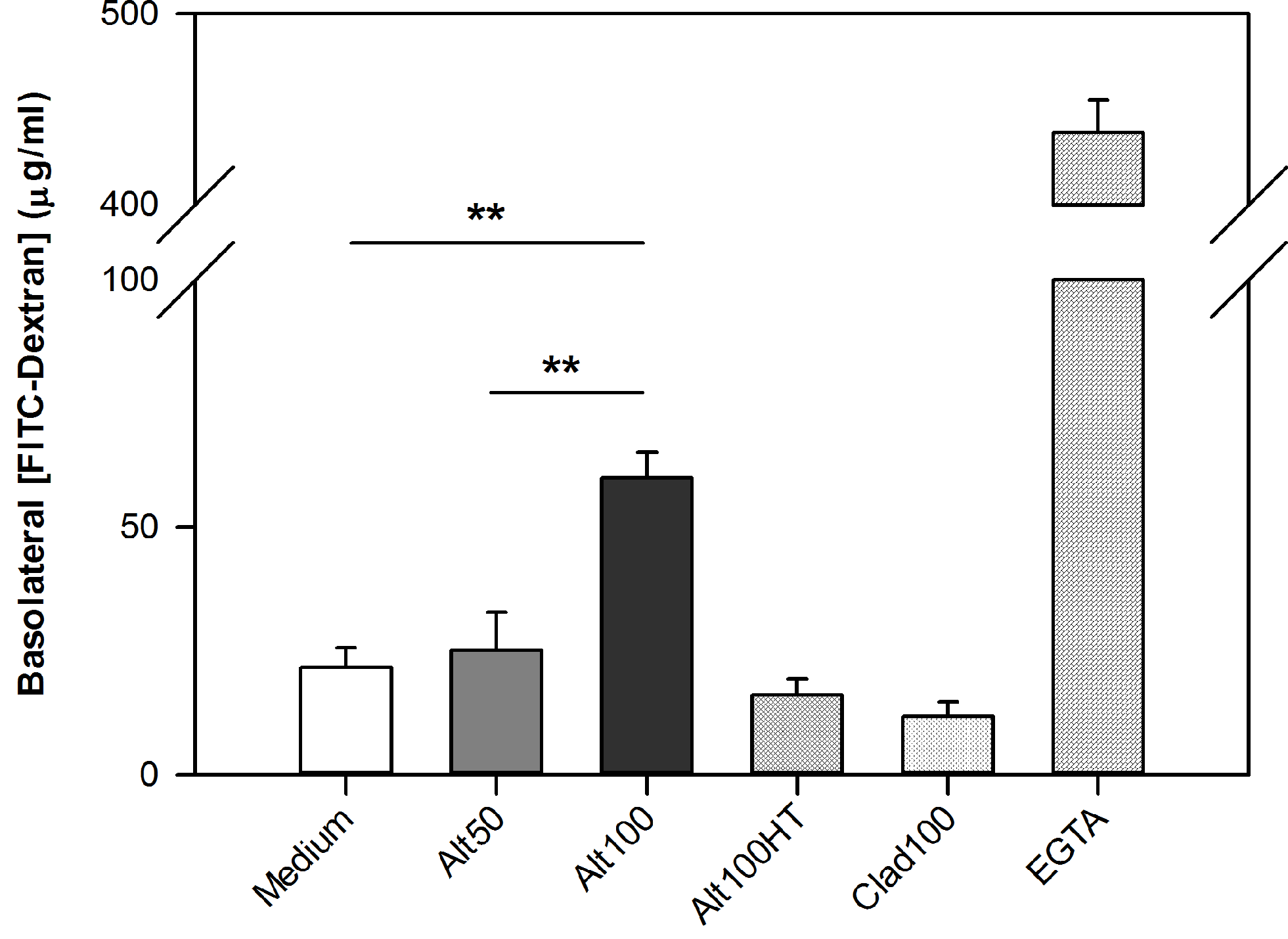

Supplement: Figure S3 — Alternaria extract increases epithelial macromolecular permeability. Polarised 16HBE cells on Transwell inserts were challenged with medium, Alt50, Alt100 (all n = 4), Alt100HT, Clad100, or EGTA (n = 2) 1 h before addition of 2 mg/ml 4 kDA FITC-dextran. After 24 h challenge, basolateral FITC-dextran concentration was determined fluorimetrically. Analysis as for Figure S1 Bars represent mean ± SEM; ** p<0.01. (TIF) [file pone.0071278.s003.tif]

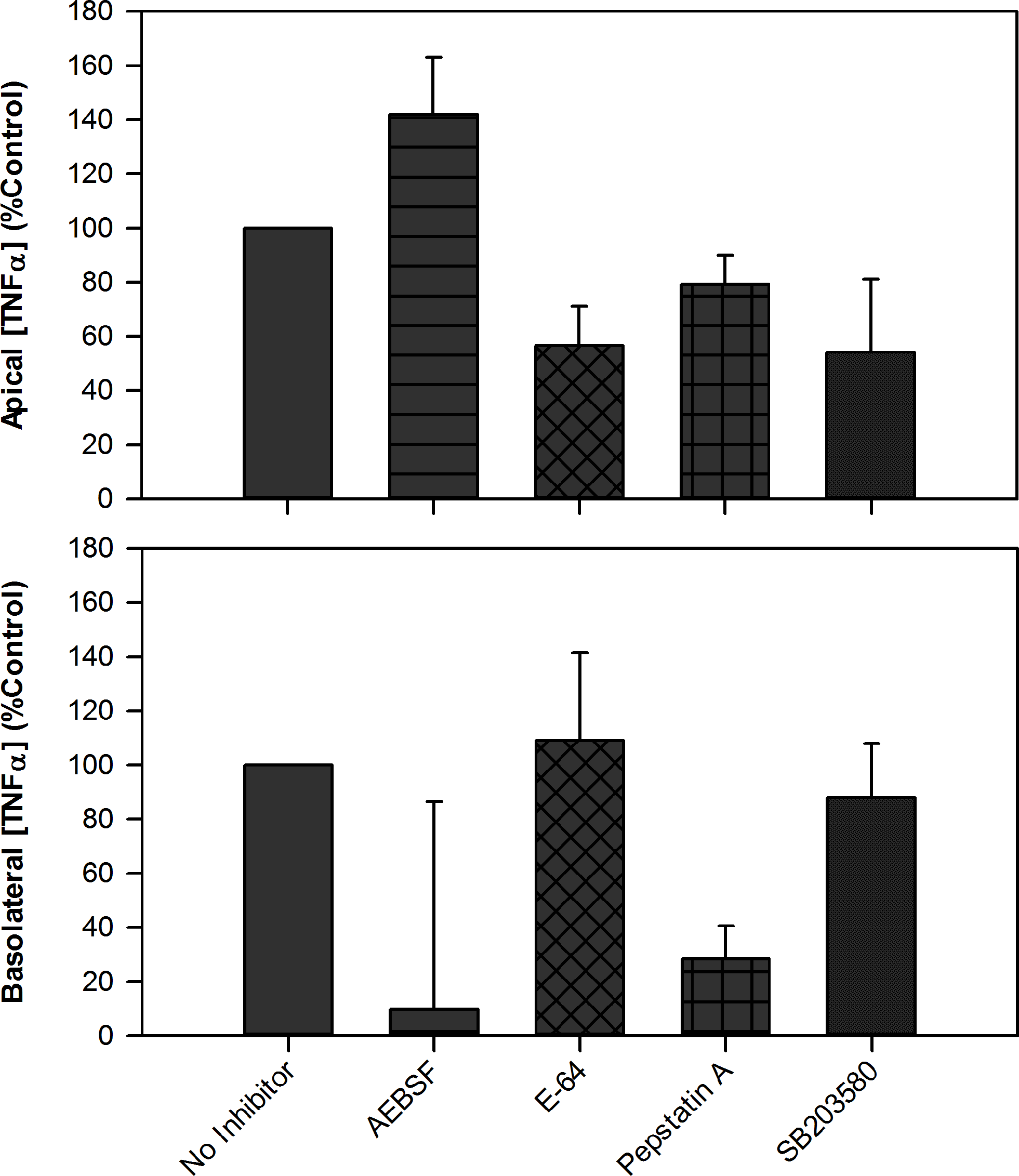

Supplement: Figure S4 — Inhibitors of proteases and p38 MAPK have no significant effect on apical or basolateral TNFα release after fungal challenge. The effect of Alternaria (100 μg/ml) on 16HBE cells was tested alone or in the presence of AEBSF (250 μM), E-64 (50 μM), Pepstatin A (0.5 μg/ml) or SB203580 (25 μM) (n = 3–8). TNFα release 24 h post-challenge was calculated as “Release (% control) = ((AltINHIB – No AltINHIB)/(AltNO INHIB – No AltNO INHIB)) ×100”, to correct for any effect of the inhibitors on baseline TNFα release without Alternaria. Analysis as for Figure S1. Data show mean ± SEM. (TIF) [file pone.0071278.s004.tif]

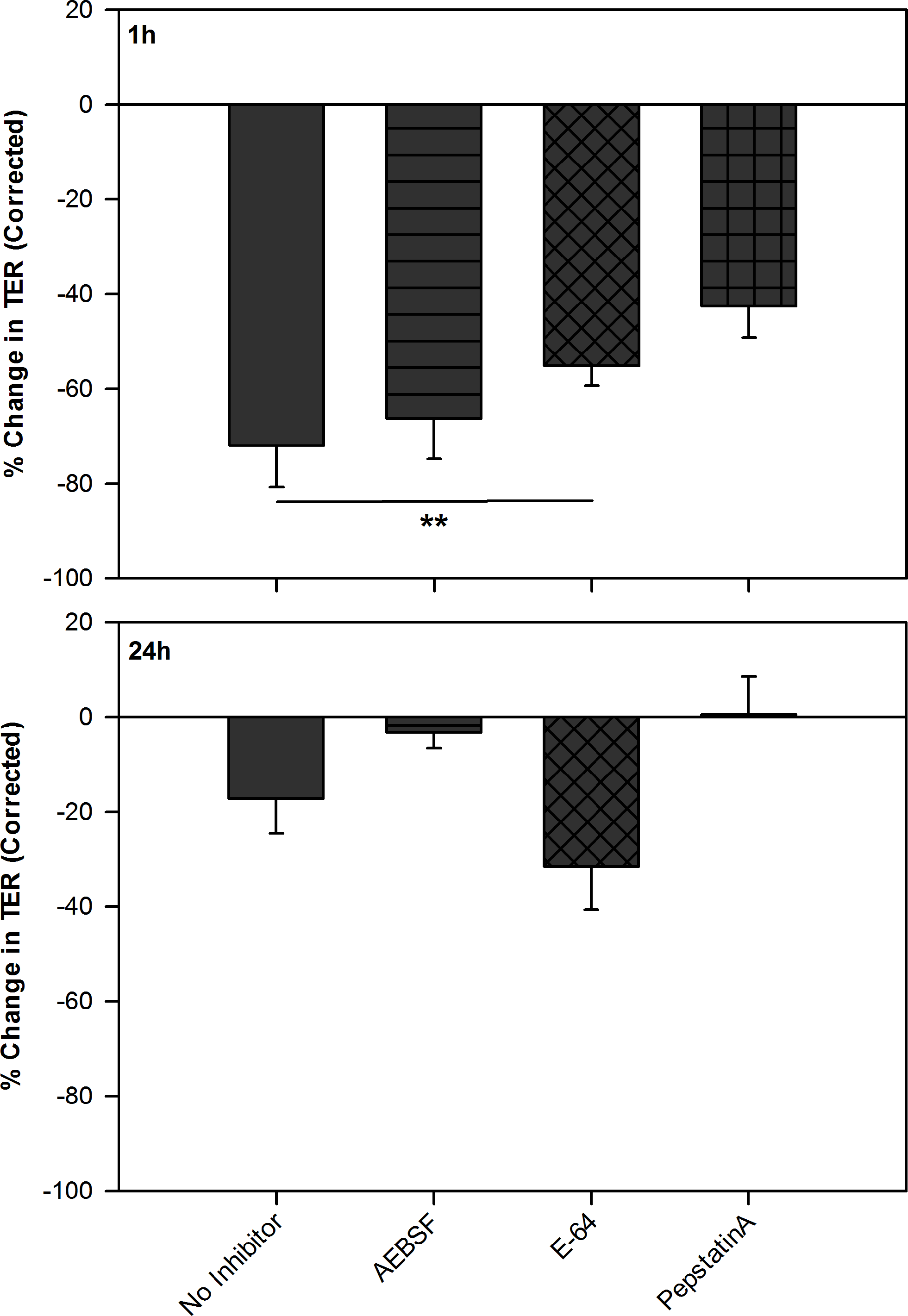

Supplement: Figure S5 — Alternaria -induced drop in TER is sensitive to inhibition of cysteine protease and p38 MAPK. The effect of Alternaria (100 μg/ml) on 16HBE cells was tested alone or in the presence of AEBSF (250 μM), E-64 (50 μM), Pepstatin A (0.5 μg/ml) or SB203580 (25 μM) (n = 3–6). TER was measured at 1 h and 24 h post-challenge, calculated as percentage change from pre-challenge, and corrected for any effect of the inhibitor alone by subtracting the percentage change in TER in the absence of Alternaria from the percentage change in TER in the presence of Alternaria, with each respective inhibitor or inhibitor-free condition. Bars represent mean change ± SEM; ** p<0.01. (TIF) [file pone.0071278.s005.tif]

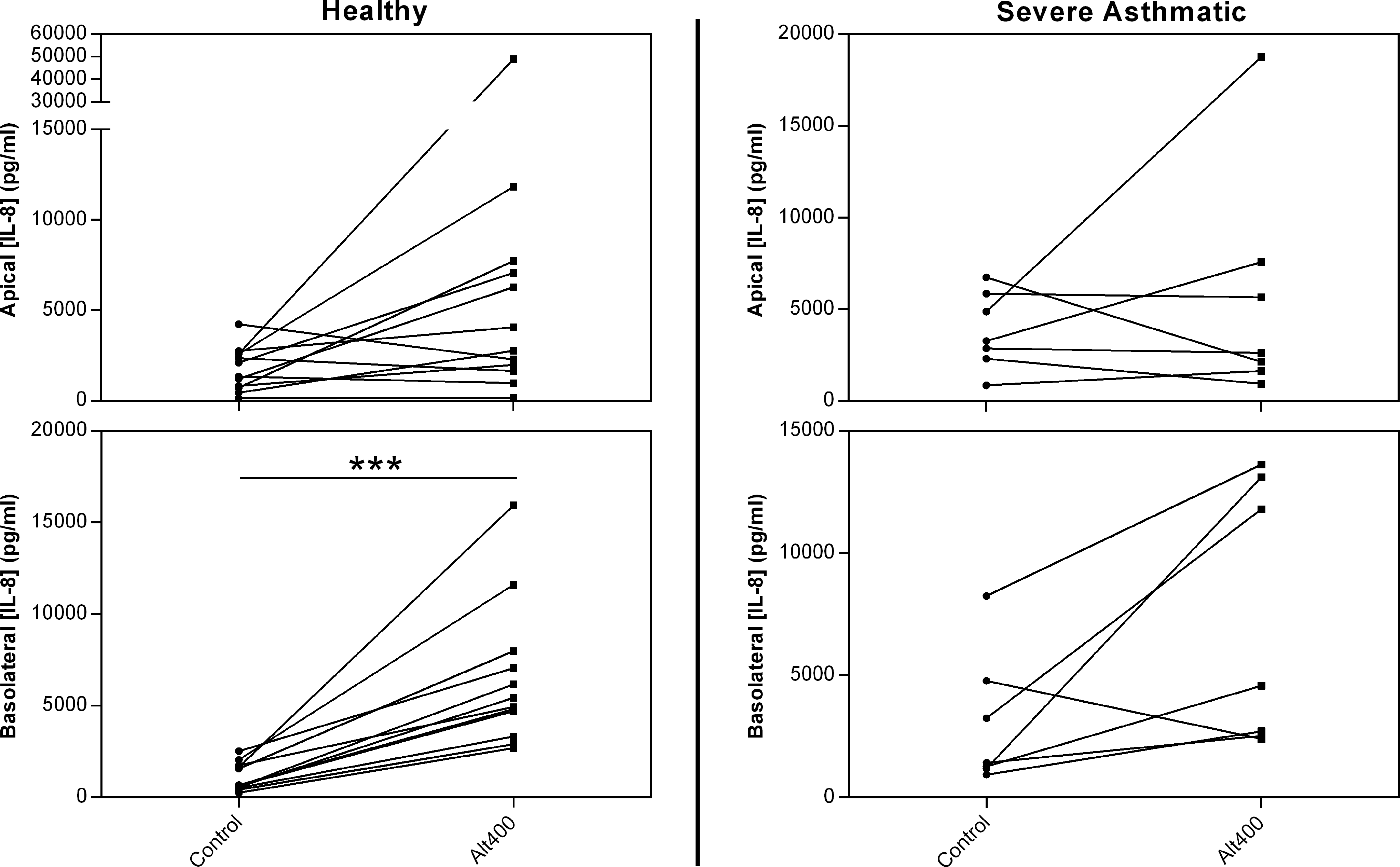

Supplement: Figure S6 — The increase in IL-8 release in healthy donor ALI cultures is driven by increased basolateral release of IL-8. ALI cultures from healthy (n = 8–12) or severely asthmatic (n = 6–7) donors were differentiated at air-liquid interface, prior to challenge with Alternaria (Alt) 400 µg/ml. IL-8 release 24 h post-challenge was determined by ELISA. Lines represent difference in individual donor cultures between control and Alt400-stimulated IL-8 release. Analysis by Wilcoxon Matched Pair test. *** p<0.001. (TIF) [file pone.0071278.s006.tif]

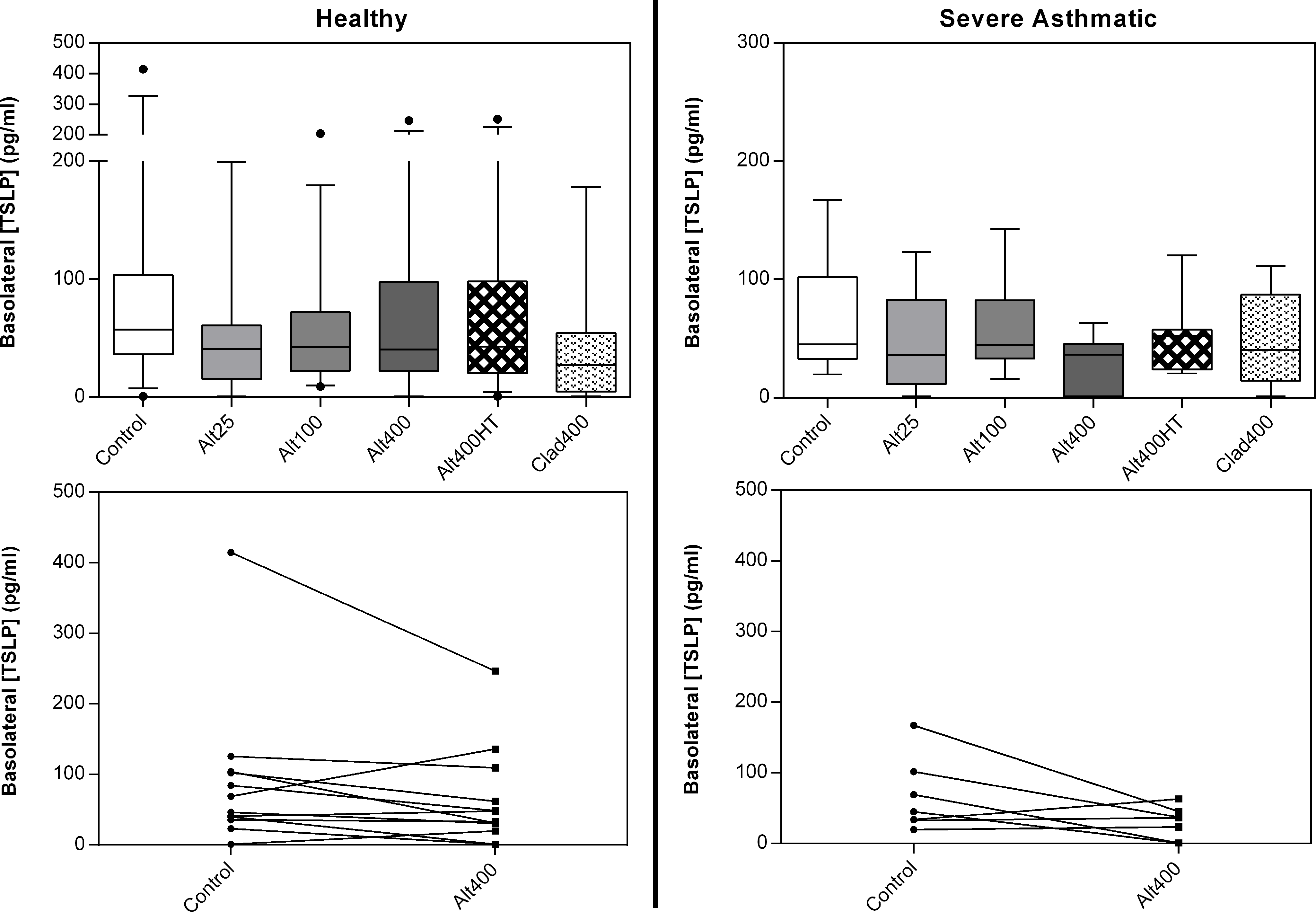

Supplement: Figure S7 — Alternaria challenge does not affect basolateral release of TSLP in healthy or severely asthmatic donor ALI cultures. ALI cultures from healthy (n = 7–12) or severely asthmatic (n = 6–7) donors were differentiated at air-liquid interface, prior to challenge with Alternaria (Alt) or Cladosporium (Clad) fungal extracts. TSLP release 24 h post-challenge was determined by ELISA. TOP: Boxes show median and 25/75th percentiles, and whiskers show 10th/90th percentiles. Analysis by Friedman's test. BOTTOM: Lines represent difference in individual donor cultures between control and Alt400-stimulated TSLP release. (TIF) [file pone.0071278.s007.tif]
