# Supplementary material for: Barrier Disrupting Effects of Alternaria Alternata Extract on Bronchial Epithelium from Asthmatic Donors
Source: PLoS One. 2013 Aug 23;8(8):e71278. doi: 10.1371/journal.pone.0071278 (PMC3751915; doi:10.1371/journal.pone.0071278)
Supplement: Information S1 — (DOCX) [file pone.0071278.s008.docx]

**SUPPORTING INFORMATION**

**BARRIER DISRUPTING EFFECTS OF *ALTERNARIA ALTERNATA* EXTRACT ON BRONCHIAL EPITHELIUM FROM ASTHMATIC DONORS**

Marina S Leino *, Matthew Loxham *, Cornelia Blume, Emily J Swindle, Nivenka P Jayasekera, Patrick W Dennison, Betty WH Shamji ^§^, Matthew J Edwards ^§^, Stephen T Holgate , Peter H Howarth, Donna E Davies

(* These authors have contributed equally to this work)

Academic Unit of Clinical and Experimental Sciences and the Southampton NIHR

Respiratory Biomedical Research Unit, University of Southampton Faculty of Medicine, Level F, Sir Henry Wellcome Laboratories, South Block, University Hospital Southampton, Tremona Road, Southampton SO16 6YD, United Kingdom and ^§^Novartis Institutes for Biomedical Research, Novartis Horsham Research Centre, Horsham, UK.

**Corresponding author:**

Matthew Loxham

The Brooke Laboratory

Mailpoint 888

University Hospital Southampton

Tremona Road

Southampton

SO16 6YD

Telephone: +44 2380 777222 x3308

Fax: +44 2380 511761

Email: m.loxham@soton.ac.uk

**Short Title:** EFFECTS OF ALTERNARIA ON BRONCHIAL EPITHELIUM

**Methods**

*Cell Culture*

The 16-HBE14o- human bronchial epithelial cell line (a gift from Professor D.C. Gruenert, San Francisco, USA) [1] was cultured in Minimal Essential Medium (MEM) with GlutaMax supplemented with 10% heat-inactivated FBS, 50 IU/ml penicillin and 50μg/ml streptomycin (all from Invitrogen, Paisley, UK) at 37ºC, 5% CO_2_. Cells were seeded at a density of 1.5 x10^5^ in 200 μl of culture medium onto polyester Transwell® membranes (0.33cm^2^ surface area, 0.4μm pore size; Corning Life Sciences, Fisher Scientific, UK) pre-coated with collagen I (Pure-Col, Nutacon BV, Leimuiden, The Netherlands). The basolateral compartment contained 500μl MEM. Medium in both compartments was changed on alternate days until confluency and showed a transepithelial electrical resistance (TER) measurement of 3000Ω cm^-2^, using an EVOM Transepithelial Voltmeter (World Precision Instruments, Sarasota, FL, USA).

Primary bronchial epithelial cells (PBECs) were grown from bronchial brushings obtained by fibre optic bronchoscopy as previously described [2] (for donor information, see Table S1). PBECs were expanded up to passage 2 before being placed at air-liquid interface (ALI) according to a previously published method [3]. Basolateral medium was changed every day except during weekends, TER was monitored every 7 days, and cultures were used for assays at day 21 when the TER was 3000Ω cm^-2^.

*Challenge*

24h prior to challenge, 16HBE cells had the apical medium replaced with serum-free MEM while the basolateral medium of ALI cultures was replaced with BEBM with 1% ITS (Sigma-Aldrich, Gillingham, UK) and 1.5μg/ml BSA. Lyophilised *Alternaria alternata* and *Cladosporium herbarum* extracts were dissolved into supplement-free medium and added apically to achieve the requisite concentrations. To assess the heat-lability of the activity in fungal extracts, aliquots of the dissolved allergen extracts were heat-treated at 65 ºC for 30 min. To delineate specific protease activity, *Alternaria* extract solution was exposed to the protease inhibitors AEBSF (500μM with *Alternaria*; 250μM final concentration on cells), E-64 (100μM; 50μM final concentration on cells), or Pepstatin A (1μg/ml; 0.5μg/ml final concentration on cells) for 30 min immediately prior to stimulation, or cells were pretreated with the p38 MAPK inhibitor SB203580 (50μM in 100μl medium applied to cells, reduced to 25μM by addition of *Alternaria* after incubation) in the apical compartment for 30 min. For 16HBE challenge, fungal extracts were applied apically in a final volume of 200μl, while 50μl was applied apically to ALI cultures to maintain the air-liquid interface. TER was measured immediately prior to the addition of extracts and thereafter at regular intervals. For TER measurements 100 μl of HBSS was added to the apical compartment immediately after harvesting of the apical supernatant, and this was removed immediately after reading of TER and discarded or combined with the harvested supernatant. At 24h, apical and basolateral media were harvested and cells were fixed for immunostaining.

*Lactate Dehydrogenase (LDH) assay*

LDH release was measured using a CytoTox 96 LDH assay kit (Promega, Southampton, UK) according to the manufacturer’s instructions. Total cellular LDH activity was determined by lysing cells with 1% Triton X-100 in culture medium for 60 min at 37°C.

*FITC-dextran passage*

FITC-dextran (4kDa) (Sigma) was added to the apical compartment at a final concentration of 2mg/ml, 1h after the addition of the fungal extracts. Basolateral FITC-dextran concentration at 24h was determined against a standard curve using a Labsystems Fluoroskan FL fluorimeter (Thermo Fisher Scientific, Waltham, MA), with excitation and emission wavelengths set to 485nm and 530nm respectively.

*Cytokine Analysis*

Release of IL-8 and TNF-α into the apical and basolateral supernatants was assayed by ELISA according to the manufacturer's instructions (R&D Systems, Abingdon, UK). For assay of TNFα release from ALI cultures, a high-sensitivity TNFα ELISA kit was used (R&D Systems). TSLP was measured using an ‘in-house’ ELISA developed by Novartis Plc (Horsham, UK), which recognises both *E. coli* expressed recombinant TSLP and naturally secreted TSLP from primary lung fibroblasts.

*Detection of Protease Activity*

*Alternaria* and *Cladosporium* extract protease activity was assessed using a protease fluorescent detection kit (Sigma-Aldrich) according to the manufacturer’s instructions. This kit was also used to measure attenuation of *Alternaria* protease activity by prior heat-treatment of the extract or the protease inhibitors AEBSF, E-64, or Pepstatin A.

*Statistical analysis*

Results were analysed by one-way repeated measures ANOVA with Bonferroni correction for pairwise analyses, or a ranked version thereof (Friedman Repeated Measures ANOVA), with Bonferroni’s or Tukey’s correction for pairwise analyses, as appropriate. All analyses were performed using SigmaPlot 11.0 (Systat Software, Hounslow, UK).

**References**

1. Cozens AL, Yezzi MJ, Kunzelmann K, Ohrui T, Chin L, et al. (1994) CFTR expression and chloride secretion in polarized immortal human bronchial epithelial cells. Am J Respir Cell Mol Biol 10: 38-47.

2. Bucchieri F, Puddicombe SM, Lordan JL, Richter A, Buchanan D, et al. (2002) Asthmatic bronchial epithelium is more susceptible to oxidant-induced apoptosis. Am J Respir Cell Mol Biol 27: 179-185.

3. Gray TE, Guzman K, Davis CW, Abdullah LH, Nettesheim P (1996) Mucociliary differentiation of serially passaged normal human tracheobronchial epithelial cells. American Journal of Respiratory Cell and Molecular Biology 14: 104-112.

**Table S1. Primary bronchial epithelial cell donor information.**

| Donor | Status | Age | Sex | FEV_1_% | Medication | Atopic |
| --- | --- | --- | --- | --- | --- | --- |
| 1 | Healthy | 19 | M | 96 | 0 | N |
| 2 | Healthy | 54 | F | 141 | 0 | N |
| 3 | Healthy | 19 | M | 104 | 0 | N |
| 4 | Healthy | 20 | F | 101 | 0 | N |
| 5 | Healthy | 20 | F | 93 | 0 | N |
| 6 | Healthy | 25 | M | 101 | 0 | N |
| 7 | Healthy | 23 | F | 120 | 0 | Y |
| 8 | Healthy | 44 | F | 88 | 0 | N |
| 9 | Healthy | 35 | M | 123 | 0 | N |
| 10 | Healthy | 19 | M | 104 | 0 | N |
| 11 | Healthy | 23 | M | 94 | 0 | N |
| 12 | Healthy | 23 | M | 106 | 0 | N |
|  |  |  |  |  |  |  |
| **Mean** |  | 27.0 | 7M/5F | 105.9 |  |  |
| **SE** |  | 3.3 |  | 4.4 |  |  |
|  |  |  |  |  |  |  |
|  |  |  |  |  |  |  |
| 13 | Severe Asthmatic | 51 | F | 76 | ICS2400, LABA | Y |
| 14 | Severe Asthmatic | 51 | F | 55 | ICS1000, LABA, Anti-Leuk | Y |
| 15 | Severe Asthmatic | 45 | M | 31 | ICS1000, LABA | Y |
| 16 | Severe Asthmatic | 34 | M | 80 | ICS3000, LABA, ANTI-LEUK | N |
| 17 | Severe Asthmatic | 45 | F | 94 | ICS3000, LABA, ANTI-LEUK | Y |
| 18 | Severe Asthmatic | 35 | F | 41 | ICS2000, LABA, ANTI-LEUK | Y |
| 19 | Severe Asthmatic | 62 | M | 34 | ICS1000, LABA, ANTI-LEUK, OCS1020MG/Y | N |
|  |  |  |  |  |  |  |
| **Mean** |  | 46.1 | 3M/4F | 58.7 |  |  |
| **SE** |  | 3.7 |  | 9.4 |  |  |

**Table S1. PBEC donor information.** Clinical characterisation of the donors of the bronchial epithelial cells used in this work. FEV_1_% - forced expiratory volume in 1 second, as a percentage of predicted value; ICS – inhaled corticosteroid (dose as equivalent to micrograms per day Beclometasone dipropionate); LABA = long acting β_2_-adrenoceptor agonist; anti-leuk = anti-leukotriene.

**Figure S1. Alternaria extract induces a heat-labile increase in TNFα release from polarised 16HBE cells.** Polarised 16HBE cells on Transwell inserts (n=3-9) were challenged apically with *Alternaria* (Alt) or *Cladosporium* (Clad) fungal extracts. Apical and basolateral supernatants were harvested 24h post-challenge. TNFα concentration was determined by ELISA. Analysis by one way repeated measures ANOVA with Bonferroni correction for pairwise analyses. Bars represent mean±SEM; ** p<0.01; *** p<0.001.

**Figure S2. *Alternaria* extract induces a dose-dependent decrease in 16HBE TER.**  TER was measured before fungal challenge of polarised 16HBE cells, and at regular intervals up to 24h thereafter (n=4-15). Graph shows TER of polarised 16HBE cultures in medium alone (▲), or with *Alternaria* extract at 50 (●) and 100 μl/ml (♦), expressed as percentage change from pre-challenge value. Points represent mean±SEM.

**Figure S3. *Alternaria* extract increases epithelial macromolecular permeability.** Polarised 16HBE cells on Transwell inserts were challenged with medium, Alt50, Alt100 (all n=4), Alt100HT, Clad100, or EGTA (n=2) 1h before addition of 2mg/ml 4kDA FITC-dextran. After 24h challenge, basolateral FITC-dextran concentration was determined fluorimetrically. Analysis as for Figure S1. Bars represent mean±SEM; ** p<0.01.

**Figure S4. Inhibitors of proteases and p38 MAPK have no significant effect on apical or basolateral TNFα release after fungal challenge.** The effect of *Alternaria* (100μg/ml) on 16HBE cells was tested alone or in the presence of AEBSF (250μM), E-64 (50μM), Pepstatin A (0.5μg/ml) or SB203580 (25μM) (n=3-8). TNFα release 24h post-challenge was calculated as “Release (% control) = ((Alt_INHIB_ – No Alt_INHIB_) / (Alt_NO_ _INHIB_ – No Alt_NO INHIB_)) x 100”, to correct for any effect of the inhibitors on baseline TNFα release without *Alternaria*. Analysis as for Figure S1. Data show mean±SEM.

**Figure S5. *Alternaria*-induced drop in TER is sensitive to inhibition of cysteine protease and p38 MAPK.** The effect of *Alternaria* (100μg/ml) on 16HBE cells was tested alone or in the presence of AEBSF (250μM), E-64 (50μM), Pepstatin A (0.5μg/ml) or SB203580 (25μM) (n=3-6). TER was measured at 1h and 24h post-challenge, calculated as percentage change from pre-challenge, and corrected for any effect of the inhibitor alone by subtracting the percentage change in TER in the absence of Alternaria from the percentage change in TER in the presence of Alternaria, with each respective inhibitor or inhibitor-free condition. Bars represent mean change±SEM; ** p<0.01.

**Figure S6. The increase in IL-8 release in healthy donor ALI cultures is driven by increased basolateral release of IL-8.** ALI cultures from healthy (n=8-12) or severely asthmatic (n=6-7) donors were differentiated at air-liquid interface, prior to challenge with Alternaria (Alt) 400µg/ml. IL-8 release 24h post-challenge was determined by ELISA. Lines represent difference in individual donor cultures between control and Alt400-stimulated IL-8 release. Analysis by Wilcoxon Matched Pair test. *** p<0.001.

**Figure S7. Alternaria challenge does not affect basolateral release of TSLP in healthy or severely asthmatic donor ALI cultures.**  ALI cultures from healthy (n=7-12) or severely asthmatic (n=6-7) donors were differentiated at air-liquid interface, prior to challenge with *Alternaria* (Alt) or *Cladosporium* (Clad) fungal extracts. TSLP release 24h post-challenge was determined by ELISA. TOP: Boxes show median and 25/75^th^ percentiles, and whiskers show 10^th^/90^th^ percentiles. Analysis by Friedman’s test. BOTTOM: Lines represent difference in individual donor cultures between control and Alt400-stimulated TSLP release.
