# Supplementary material for: Barrier Disrupting Effects of Alternaria Alternata Extract on Bronchial Epithelium from Asthmatic Donors
Source: PLoS One. 2013 Aug 23;8(8):e71278. doi: 10.1371/journal.pone.0071278 (PMC3751915; doi:10.1371/journal.pone.0071278)
Supplement: Table S1 — PBEC donor information. Clinical characterisation of the donors of the bronchial epithelial cells used in this work. FEV1% – forced expiratory volume in 1 second, as a percentage of predicted value; ICS – inhaled corticosteroid (dose as equivalent to micrograms per day Beclometasone dipropionate); LABA = long acting β2-adrenoceptor agonist; anti-leuk = anti-leukotriene. (DOCX) [file pone.0071278.s009.docx]

**Table S1. Primary bronchial epithelial cell donor information.**

| Donor | Status | Age | Sex | FEV_1_% | Medication | Atopic |
| --- | --- | --- | --- | --- | --- | --- |
| 1 | Healthy | 19 | M | 96 | 0 | N |
| 2 | Healthy | 54 | F | 141 | 0 | N |
| 3 | Healthy | 19 | M | 104 | 0 | N |
| 4 | Healthy | 20 | F | 101 | 0 | N |
| 5 | Healthy | 20 | F | 93 | 0 | N |
| 6 | Healthy | 25 | M | 101 | 0 | N |
| 7 | Healthy | 23 | F | 120 | 0 | Y |
| 8 | Healthy | 44 | F | 88 | 0 | N |
| 9 | Healthy | 35 | M | 123 | 0 | N |
| 10 | Healthy | 19 | M | 104 | 0 | N |
| 11 | Healthy | 23 | M | 94 | 0 | N |
| 12 | Healthy | 23 | M | 106 | 0 | N |
|  |  |  |  |  |  |  |
| **Mean** |  | 27.0 | 7M/5F | 105.9 |  |  |
| **SE** |  | 3.3 |  | 4.4 |  |  |
|  |  |  |  |  |  |  |
|  |  |  |  |  |  |  |
| 13 | Severe Asthmatic | 51 | F | 76 | ICS2400, LABA | Y |
| 14 | Severe Asthmatic | 51 | F | 55 | ICS1000, LABA, Anti-Leuk | Y |
| 15 | Severe Asthmatic | 45 | M | 31 | ICS1000, LABA | Y |
| 16 | Severe Asthmatic | 34 | M | 80 | ICS3000, LABA, ANTI-LEUK | N |
| 17 | Severe Asthmatic | 45 | F | 94 | ICS3000, LABA, ANTI-LEUK | Y |
| 18 | Severe Asthmatic | 35 | F | 41 | ICS2000, LABA, ANTI-LEUK | Y |
| 19 | Severe Asthmatic | 62 | M | 34 | ICS1000, LABA, ANTI-LEUK, OCS1020MG/Y | N |
|  |  |  |  |  |  |  |
| **Mean** |  | 46.1 | 3M/4F | 58.7 |  |  |
| **SE** |  | 3.7 |  | 9.4 |  |  |

**Table S1. PBEC donor information.** Clinical characterisation of the donors of the bronchial epithelial cells used in this work. FEV_1_% - forced expiratory volume in 1 second, as a percentage of predicted value; ICS – inhaled corticosteroid (dose as equivalent to micrograms per day Beclometasone dipropionate); LABA = long acting β_2_-adrenoceptor agonist; anti-leuk = anti-leukotriene.
